# Modulation of allosteric coupling by mutations: from protein dynamics and packing to altered native ensembles and function

**DOI:** 10.1016/j.sbi.2018.09.004

**Published:** 2018-09-28

**Authors:** Athi N Naganathan

**Affiliations:** Department of Biotechnology, Bhupat & Jyoti Mehta School of Biosciences, Indian Institute of Technology Madras, Chennai 600036, India

## Abstract

A large body of work has gone into understanding the effect of mutations on protein structure and function. Conventional treatments have involved quantifying the change in stability, activity and relaxation rates of the mutants with respect to the wild-type protein. However, it is now becoming increasingly apparent that mutational perturbations consistently modulate the packing and dynamics of a significant fraction of protein residues, even those that are located >10–15 Å from the mutated site. Such long-range modulation of protein features can distinctly tune protein stability and the native conformational ensemble contributing to allosteric modulation of function. In this review, I summarize a series of experimental and computational observations that highlight the incredibly pliable nature of proteins and their response to mutational perturbations manifested via the intra-protein interaction network. I highlight how an intimate understanding of mutational effects could pave the way for integrating stability, folding, cooperativity and even allostery within a single physical framework.

## Introduction

Mutations in proteins occur via multiple well-understood molecular mechanisms primarily at the level of DNA contributing to variability in the population. Such variability is the cornerstone of evolution as functionally advantageous mutations get fixed in the presence of a selection pressure. Decades of work on mutations have revealed rich information on protein conformational behavior, binding site identities and thermodynamics, folding mechanisms and allostery. In parallel, understanding and modeling mutational effects has tremendous implications in not just designing proteins with enhanced solubility, stability, and catalytic efficiency, but also to understand evolutionary trajectories of proteins, enzyme evolvability and the contribution of mutant phenotypes to organismal fitness [1–5]. Numerous avenues are currently available to engineer proteins ranging from charge-charge interactions on the protein surface [6] to directed evolution [7] and saturation mutagenesis-based approaches [8,9].

A ‘neutral’ mutation is conventionally defined as a perturbation that has little effect on the organismal fitness (say, functioning of a protein or survival of the organism). However, the same mutation in conjunction with mutations at other sites can have a positive or a negative effect on the fitness landscape. How is this epistatic characteristic enabled? It is important to realize that the interior (surface) of a protein is a highly unique environment determined by the unique protein sequence with specific packing (electrostatic/polar) interactions. Therefore, any perturbation from a mutation is expected to be complex, as it would involve an abrupt reorganization of the evolutionarily tuned interaction network. In fact, it is well recognized in the field of protein NMR that mutations manifest as non-trivial effects on chemical shifts and order parameters of a majority of residues in the protein. However, there is an apparent disconnect between an NMR view of mutational effects (complex changes in multiple terms) and studies that merely quantify the change in stability or folding rate of the mutant compared to the wild-type. In this review, I highlight and summarize some of the recent developments towards resolving this apparent conflict and how a mere consideration of the intra-protein interaction network provides a convincing rationale with several testable predictions.

## Mutational effects propagate beyond the firstshell of interactions

**Figure 1:**
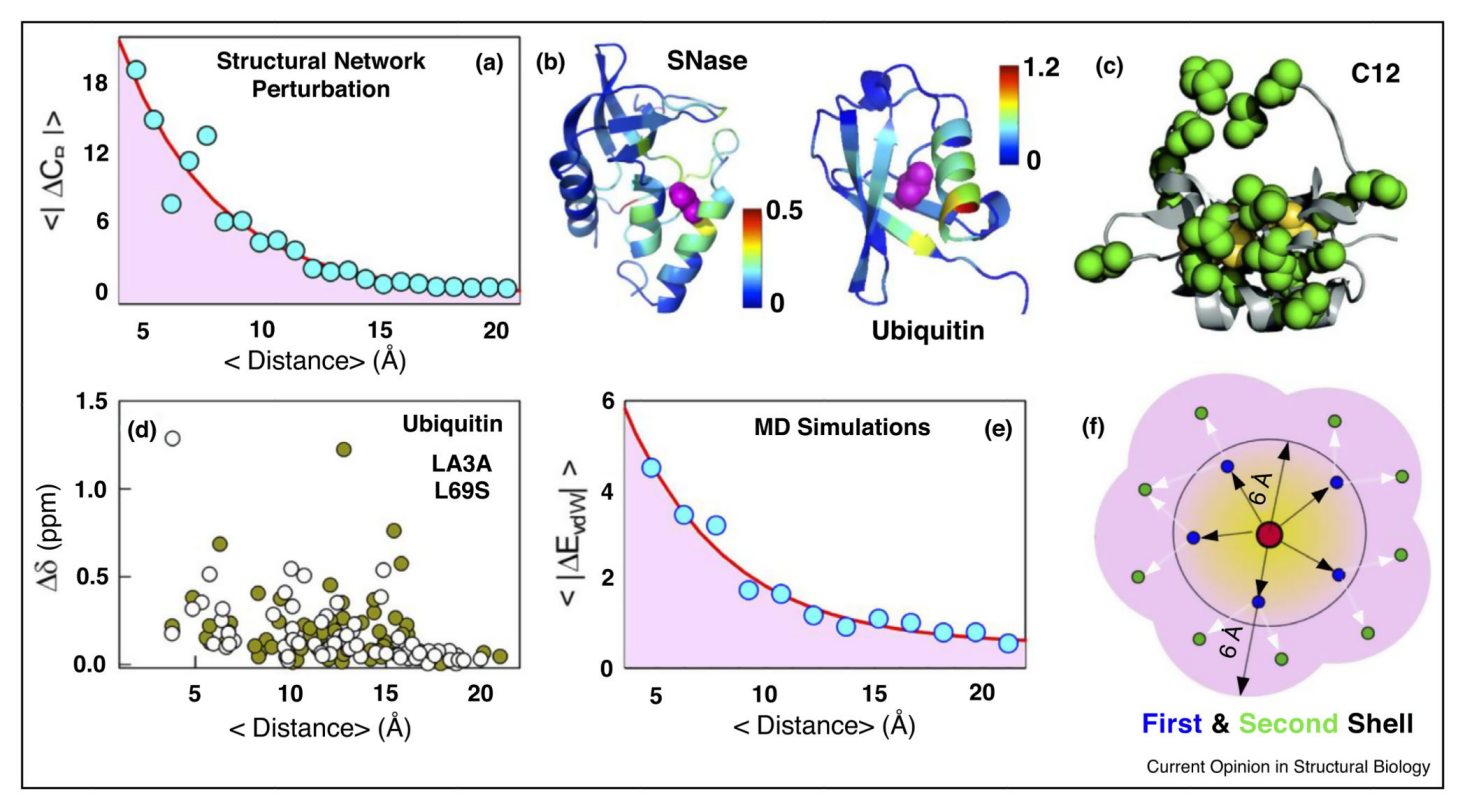
Mutational effects are consistently felt over long distances with evidence available from varied approaches. **(a)** Average changes in network connectivity, quantified by betweenness centrality (C_B_), plotted as a function of mean C*_α_*-C*_α_* distance from the mutated site from a ‘toy’ model of Ubiquitin that treats protein residues as nodes and their interactions as edges [11•]. **(b)** Experimental chemical shift perturbations on mutations mapped on to the structure of SNase (L125A; [17•]) and Ubiquitin (L43A; [14]), respectively. **(c)** Residues whose NMR order parameters are affected (green) on core mutations of residues shown in yellow in CI2 [13]. Reprinted with permission from Ref. [13], Copyright (2008), American Chemical Society. **(d)** Experimental chemical shift perturbations as a function of distance from the mutated site for specific ubiquitin mutations [11•,25•]. **(e)** Mean absolute changes in van der Waals packing interactions (ordinate in kJ mol^–1^) for seven core substitutions in ubiquitin plotted as a function of distance from the mutated site from all-atom MD simulations (circles) together with an exponential fit (red) [11•]. **(f)** A cartoon representation of how perturbation of a residue (red) affects not only its first-shell neighbors (blue and interactions as black arrows) but also the neighbors of neighbors (green and interactions as white arrows).

A reductionist approach in viewing proteins is to treat protein residues as nodes and the intra-protein interactions as edges [10]. When the edges are perturbed, either by deletion or by modulation of their strengths, the network properties are modulated not just in the immediate neighborhood but also at longer distances [11•] (Figure 1a). Such propagation and dissipation (used strictly in an equilibrium sense with no kinetic connotation) of perturbations with increasing distances from the source of perturbation is a robust feature of networks [12]. True to this expectation, point mutations in CI2 [13], Ubiquitin [14], T4 lysozyme [15], SSo7d [16], Staphylococcal nuclease [17•] and Protein L [18] all contribute to persistent modulation of chemical shifts, hydrogen-exchange protection factors or dynamics almost throughout the entire structure (for example, see Figure 1b, c). Such long-distance coupling of residues can affect catalysis in enzymes as shown through single-point and double-point mutations (>15 Å from the active site) in dihydrofolate reductase that modulate the rate of hydride transfer by up to three orders of magnitude [19,20]. By measuring chemical shift perturbations (CSPs) of β_1_-adrenergic receptor upon mutations and ligand binding, Grzesiek and coworkers identified that both the structural perturbations propagate to long distances determining the functionally relevant motions of the transmembrane helices [21•]. A study on Cyclophilin A, a peptidyl prolyl cis–trans isomerase, reported a 30% reduction in the rate of isomerization upon a conservative V29L mutation located nearly 15 Å away from the active site [22•]. While the activity modulation is minor in Cyclophilin, a 1000-fold increase in phosphotriesterase activity could be engineered in a bacterial lactonase by ‘tinkering’ with mutations in the second-shell of the active-site residues [23•]. Such extreme behaviors and context-dependence could be a manifestation of the robustness of the intra-protein interaction network to perturbations wherein the network readjusts to accommodate a residue by altering the dynamics and packing of distant residues. It also suggests that multiple mutations might be required in a protein-specific manner to irreversibly alter the interaction network or the correlated motions and hence the functional output.

The fact that distal mutations can alter activity (to variable extents) raises questions on whether this is an intrinsic response of proteins to structural perturbations due to the fluid-like nature of the protein interior [24]. True to this expectation, a global analysis of 25 mutations from 12 different protein structures from the viewpoint of chemical shift perturbations revealed that the effect of mutations could be consistently felt even up till 10–20 Å from the mutation site, and is independent of the nature of the mutation, protein type or secondary structure content (for example, see Figure 1d) [25•]. Experimental double-mutant cycles that measure the degree of energetic coupling of one reside to another reveal a similar long-range coupling of residues [26,27]. In all of the cases above, an exponential-like dissipation of network parameters, coupling energy or chemical shift perturbations have been identified highlighting the possibility of a universal function form to describe mutational effects [25•]. The pervasive long-range coupling patterns explain the large conservation of even distal residues (as far as 20–27 Å) around the active site of enzymes [28•]. It is important to emphasize here that such conservation can have varied origins including functional requirements (either at the active site or binding of an effector at an allosteric site), stability (thermodynamic and kinetic), preventing aggregation and so on. As an aside, I would like to point out that long-range structural modulation is not only observed on mutational perturbations, but also on ligand-binding and phosphorylation [25•], similar to the domino-like propagative-cum-dissipative phenomenon observed in repeat proteins [29].

## Truncation mutations primarily weaken native interactions

What are the molecular origins of destabilization induced by truncation mutations? An analysis of microsecond-long molecular dynamics trajectories of Ubiquitin WT and seven aliphatic truncation mutations revealed a distinct weakening of packing interactions across nearly the entire protein. The relative residue-level van der Waals interaction energy approaches zero (i.e. no perturbation) only at longer distances from the mutated site (Figure 1e) [11•]. Taking a cue from MD simulations, the effect of truncation mutations was recently modeled by considering two shells of interactions around the perturbed residue [11•]; the first shell accounts for the neighbors within a 6 Å distance from the mutated site, while the second shell accounts for the neighbors of neighbors and thus residues nearly 12 Å from the mutated site. By introducing different destabilization magnitudes in the first- and second-shells, accounting for the nature of mutation and parameterizing them into a statistical mechanical model [30,31], it was possible to reproduce the changes in stabilities of 375 truncation mutations in 19 different proteins with a correlation and slope comparable to that from the multi-parameter FOLDX energy function [11•]. In other words, on accounting for the nature of the mutation (merely from the ratio of atoms in the mutant compared to the WT), the first- and second-shell van der Waals interactions are weakened by 50% and 20%, respectively (Figure 1f). However, since there are many more interactions that define the second-shell, the energetic contributions are near equivalent but distributed throughout the structure. It is important to emphasize the implication of the above statement; for instance, L43, which is located in the hydrophobic core of ubiquitin is therefore connected to ~80% of the 76 residues in ubiquitin, underscoring the extent to which the intraprotein interaction network can be perturbed.

While the discussion above is primarily on truncation mutations, mutations that enhance the molecular volume also decrease protein stability though the mechanism is still unclear. Since the interior of a protein displays the packing density of a solid (despite displaying large dynamics [24,32]), any change in the protein interior environment (that is unique as the sequences themselves are unique) would disrupt interactions, as the protein molecule would struggle to fit in an amino acid with a larger volume. Not surprisingly, mutations that enhance molecular volume also contribute to an exponential-like dependence of the chemical shift perturbations as a function of distance from the mutated site [25•]. The same applies to mutations to glycine that dramatically enhance the basal backbone fluctuations (~4 kJ mol^–1^ destabilization at 310 K from merely single-site backbone entropy considerations [33]) and could manifest as nontrivial effects on the folding-functional landscape.

## Population redistribution, partial unfolding and shifts in the native ensemble

**Figure 2:**
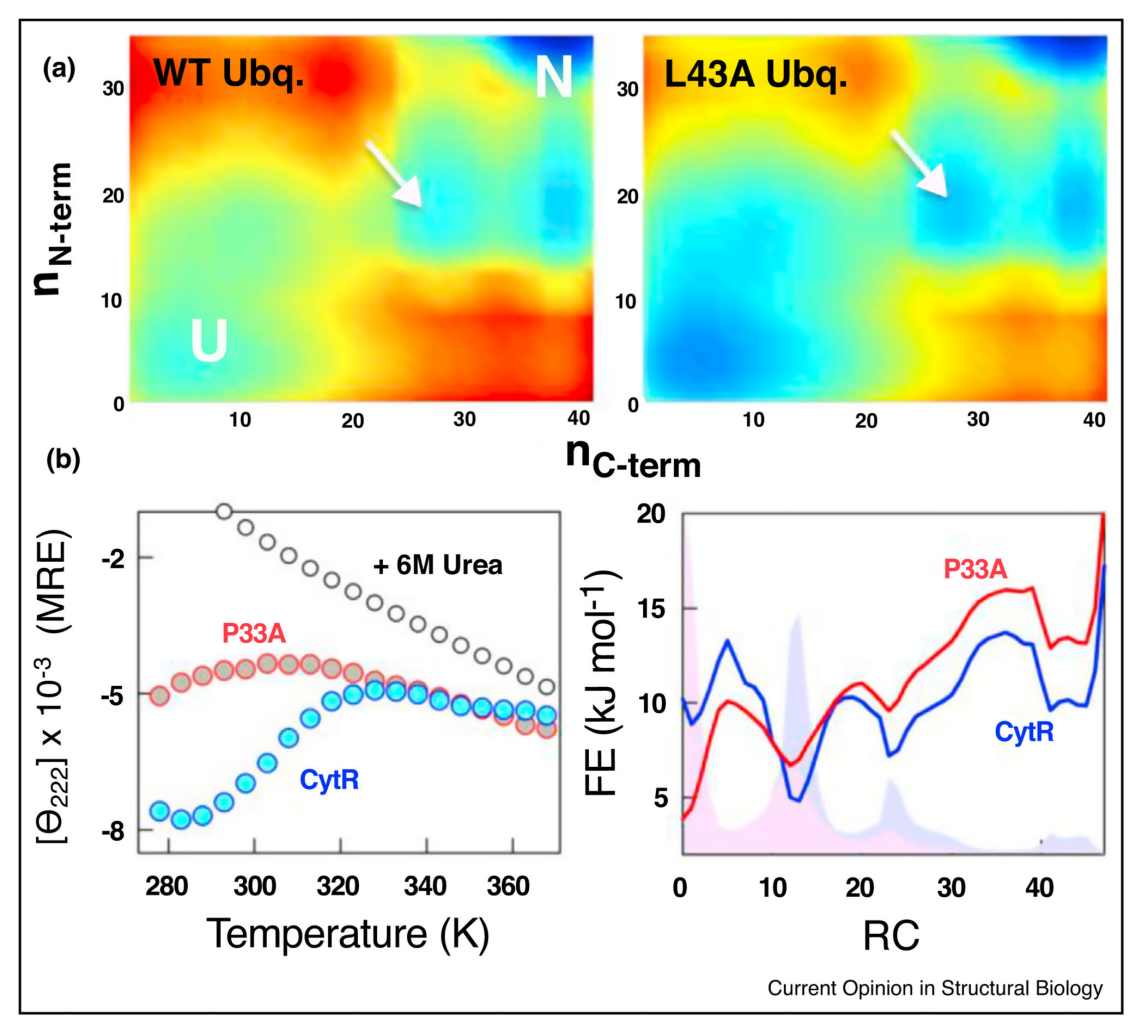
Stability changes and modulation of the folding-function landscape. **(a)** Projection of conformations onto a two-dimensional landscape generated from the WSME model for WT ubiquitin and its mutant L43A. n_N-term_ and n_C-term_ represent the number of residues structured in the N- and C-terminii, respectively. The arrow points to intermediate-like states in the landscape that are stabilized on mutations (N stands for native and U for unfolded macrostates). **(b)** (Left Panel) Changes in the secondary-structure upon mutating a proline to alanine (P33A) in a disordered protein CytR. Note that the proline is present in the loop region connecting two helices and not nucleating a helix. (Right Panel) One-dimensional free energy profiles as a function of the number of structured residues as the reaction coordinate (RC). The landscape is non-trivially modified with the population of a folded-like excited state decreasing on proline substitution [46].

Protein native states are accurately defined as ensembles of multiple conformations or substates that are critical for function. Thermodynamic fluctuations are a feature intrinsic to polymeric protein chains arising from the finite size of protein molecules and the weak nature of the non-covalent interactions [34]. From a statistical mechanical perspective, it can therefore be immediately recognized that upon mutation the statistical weights and hence the probabilities of all the conformations in which the residue is structured would be modulated. This in turn would manifest as shifts in the distribution of conformations in the native ensemble and enhanced population of intermediate or excited states apart from higher unfolded state populations (Figure 2a). This immediately explains why mutational approaches to enhancing excited state populations or intermediates have been immensely successful as in the studies involving T4 lysozyme and Fyn-SH3 domains [15,35]. Similar mutation-induced population redistributions have also been reported in GPCRs [36,37], CAP [38], Ubiquitin [39•], U1A [40], Cyclophilin [22•] and Adenylate kinase [41], with distinct effects on function in each case.

Redistribution of populations in the native ensemble tune functions and downstream signaling responses revealing avenues by which molecular responses to environmental variables could be acquired, the molecular mechanisms of drug-resistance and onset of disease conditions. Work on p97 ATPase and NAD(P)H: quinone oxidoreductase 1 (NQO1) highlight that disease causing mutations shift the conformational substates in a graded manner thus compromising innate activity [42•,^43^]. Similarly, distal mutations on tryptophan synthase shift the conformational ensemble to the extent of modulating the rate-limiting catalytic step [44]. Recently, destabilizing distal and surface glycine mutations on Adenylate Kinase (AK) have been shown to influence both enzyme activity and substrate affinity [45•]; these mutations destabilize the native ensemble through partial unfolding of AK domains shedding light on how enzymes from psychrophilic organisms could tune their basal activity to compensate for lower thermal energy.

Note that such population redistributions upon mutations need not be restricted to folded proteins but even intrinsically disordered proteins. The outcome of such mutations in disordered proteins is expected to be non-trivial due to the heterogeneous nature of IDP ensembles manifesting as large signal changes in equilibrium [46,47] (Figure 2b), binding affinity [46–49], association and dissociation rate constants [48–51], altered binding transition-state ensembles [50] and even induce liquid-to-solid phase transition [52].

## Allosteric mechanisms and paths from mutational perturbations?

It is well established that protein residues are coupled to each other through both the hydrogen-bond network and packing interactions leading to correlated motions or fluctuations in equilibrium that are critical for function. Such intra-molecular interaction-networks serve as channels for signal transmission playing a prominent role in dynamic allostery [53–55] (modulation of activity without changes in the overall structure upon distal perturbations). Since mutational effects are not localized it is natural to expect that they also modulate the communication network and hence influence function. In fact, a wide range of mutational tolerance and non-trivial functional outcomes has been observed in ubiquitin [56] and light-oxygen-voltage domain 2 [57]. Mutational studies on PDZ report that nearly the entire protein interaction network has evolved towards optimizing activity and potentially to minimize cross-reactivity while uncovering allosteric singling paths [58,59]. Similarly, extensive alanine-scanning mutagenesis has revealed that a significant fraction of residues (~30%) in the human liver pyruvate kinase can influence the binding to its substrate, PEP, in the presence of the activator Fru-1, 6-BP [60•].

**Figure 3:**
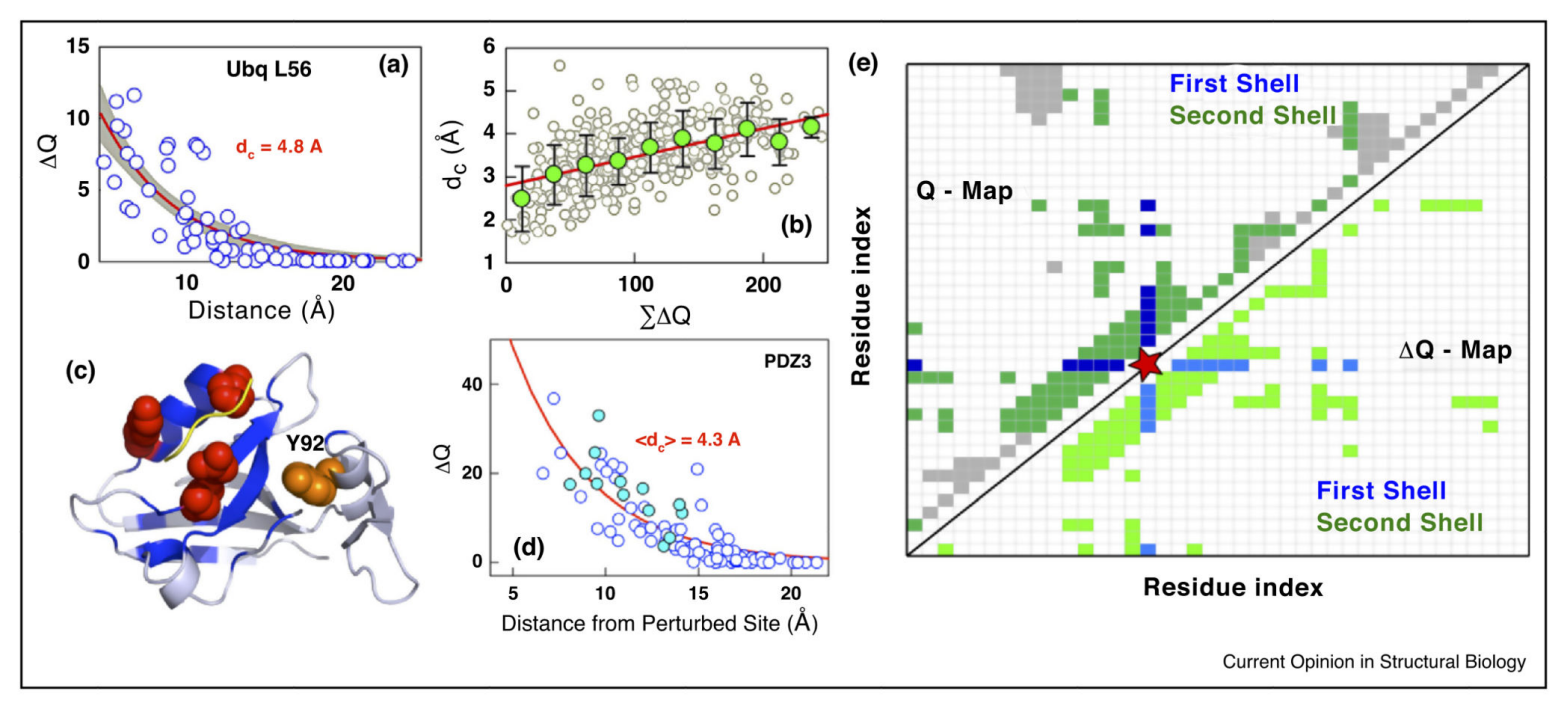
A structural perturbation approach (SPA), an *in silico* version of alanine-scanning mutagenesis, towards understanding allosteric coupling [62•]. **(a)** Changes in packing density (∆Q) as a function of distance upon L56A substitution in ubiquitin extracted through the SPA. The red curve is a single-exponential fit highlighting a coupling distance (d_C_) of 4.8 Å. **(b)** The coupling distance is approximately linearly related (red line) to the overall perturbation magnitude (abscissa). This indicates that larger residues that are located in the protein interior are extensively coupled, the perturbation of which can contribute to significant changes in packing density and hence dynamics-folding-function behaviors. The gray circles are from a perturbation analysis of all residues in six different proteins while the green circles are block averages. **(c)** Perturbation of the residues in red in PDZ3 reveals strong coupling to several residues (blue) that can potentially modulate the binding of the peptide (yellow). Note that Y92, a PTM site, is located in vicinity of the perturbed residues indicating how information on PTM could be transmitted to the binding site. **(d)** An SPA of the three residues shown in red again results in an exponential dependence of ∆Q (blue circles). The coupled residues identified using the statistical coupling analysis (SCA) is shown as filled circles are in good agreement with that predicted from the SPA. **(e)** A schematic of how longrange coupling can be extracted from the SPA. The perturbed residue is shown in red, unperturbed residues in gray, the contact map as the upper left triangular matrix and the ∆Q-map as the lower right triangular matrix. On perturbing a residue in red, apart from the first-shell neighbors (dark blue), the second shell is also affected (dark green) that constitutes a significant fraction in a small single-domain protein. The effective number of interactions that are lost are shown in the ∆Q-map (a uniform coloring code is employed for the sake of clarity).

Since the precise mechanistic details of allostery are yet to be completely established with the possibility of large context dependence, a whole-protein mutagenesis (alanine-scanning, for example) might be a way forward to test mechanistic models without preconceived notions or biases in the analysis, as also argued for in a recent work [61]. An *in silico* version of this is the structural perturbation approach (SPA; [62•]) wherein every residue is mutated to alanine or glycine (perturbation) and the immediate environment is probed for the presence of strong packing (also see [63,64] for similar methods). Following this, two parameters are extracted for every residue — the coupling distance (d_C_) and the total perturbation (ΣΔQ) — that provide information on the extent to which the perturbed residue is coupled to its neighbors (Figure 3a, b). Interestingly, the SPA reproduces the results of statistical coupling analysis (SCA [65]) with just a single structure as input and from mere distance considerations (Figure 3c, d) while revealing additional allosteric sites that could be experimentally tested [62•].

Deducing allosteric communication paths is almost entirely the purview of computational methods [66–71]. Since signal transmission should occur through the network of non-covalent interactions in the protein interior (any surface propagation would be quickly damped by solvent collisions) it is necessary that such methods also reproduce mutational destabilization thermodynamics. This would provide an independent test for the relative strengths of non-covalent interactions at different regions of the protein structure required to model signal propagation as put forth by Ernesto Freire [72]: “ … the propagation of binding signals should obey precise thermodynamic rules, and the location of allosteric sites should be dictated by thermodynamic stability criteria within the protein.” The overall features of the signaling paths should not only be consistent with the available chemical shift perturbations upon mutations (at least at the level of relative trends) but also the mutation-induced destabilization providing a sound equilibrium-thermodynamic framework for modeling allosteric communication networks.

## On folding mechanisms and cooperativity

Putative transition state structures and hence protein folding mechanisms are generally inferred from Φ-value analysis that involves measuring the changes in stability and (un)folding rates upon point mutations [73]. One of the primary assumptions of this approach is that the recommended truncation mutations in the protein interior [73] influence only the nearest neighbors (or the first-shell) providing an intimate view of the degree of structure in the transition state. Given that mutational effects consistently propagate beyond the first shell of interactions and modulate both packing and dynamics of distant residues, it is likely that Φ-values represent an effective average of multiple energetic and entropic terms and not just the extent of local structure. The frequently observed folding Φ-value of ~0.3 (independent of protein type, structure or mutation [74]) could therefore represent the fraction of stabilization free energy gained during folding [75,76]. It remains to be seen if it is possible to disentangle the energetic and structural contributions to Φ-values from computational studies. Moreover, a two-state-like treatment does not account for altered dynamics or population redistributions within the native well, necessitating a shift towards the use of more detailed thermodynamic models that could potentially provide exciting insights.

Cooperativity is feature intrinsic to systems held together by weak non-covalent interactions, and in proteins it is quantified in terms of the slope of the unfolding curve, folding barrier heights or other extent of similarity of atomic-level unfolding curves [77–79]. Cooperativity features are thus intrinsically related to the contact environment of residues in the protein. Therefore, it should be possible to perform a series of alanine-scanning experiments and iteratively identify the extent to which the interaction shell radius (or the coupling distance [11•,25•,62•]; Figure 3a) needs to be modified to account for the destabilization thermodynamics. In fact, the structural perturbation approach (SPA) can be extended to generate a ΔQ-map (similar to the contact map, or Q-map; Figure 3e) that highlights the degree of coupling of every residue with its neighbors [62•]; such maps emphasize that second-shell interactions around a residue should be formed to consider the residue to be ‘folded’ (Figure 3e). It remains to be seen if such perturbation-based approaches alone are sufficient to generate cooperativity indices or regions of structure that are more coupled (or more locally stable and hence more cooperative) than others.

## Concluding remarks

Recent experiments and computational works highlight that mutations in the protein interior manifest as altered chemical-shifts, order parameters (dynamics), HX protection factors, and modulation of packing interactions involving a significant fraction of protein residues. Accordingly, mutational perturbations are better understood in terms of their impact on the underlying interaction network or correlated motions and likely serve as the evolutionary first-step towards altered protein activity, diseased states, functional promiscuity and fold-switching. Given the robustness of the interaction network to mutations, successful engineering of enzymatic activity might require multiple perturbations [80–82] while in some cases single or double-mutations alone have been successful highlighting a certain degree of context-dependence. Deciphering this context dependence could be the way forward to engineer protein function at will.

Surface mutations also contribute to complex alteration of folded and unfolded ensembles apart from folding mechanisms [83–86]. It is therefore possible that even apparently neutral mutations modulate specific features of the native conformational ensemble which is however invisible or challenging to identify in the absence of a functional output. These observations underline the need to expand the outlook on mutational outcomes to include perturbation of native conformational ensembles, populated intermediate- and excited-states and redistribution of dynamics. Since mutations alter the evolutionarily constrained intra-molecular network of interactions, they are also expected to reshape the folding funnel and hence tune folding mechanisms, aspects that could be explored with advanced computational protocols and experiments. However, there is still a distance to travel in understanding the impact of mutations at a distal site in more functional terms — will a specific mutation at a distant site enhance or diminish binding affinity to the substrate? Detailed and intimate characterization of mutational effects supplemented with quantitative modeling could thus open up new vistas with implications in protein design, function and allostery.
